# Adjuvant Radiation Therapy in Macroscopic Regional Nodal Melanoma

**DOI:** 10.3390/cancers16233950

**Published:** 2024-11-25

**Authors:** Gerald B. Fogarty

**Affiliations:** 1 Icon Cancer Centre, 1-3 Macarthur Ave, Revesby, NSW 2022, Australia; gerald.fogarty@icon.team; 2 Sydney Brachytherapy Group, 156/158 Pacific Hwy, Greenwich, NSW 2065, Australia; 3 Faculty of Science, University of Technology Sydney, 15 Broadway, Ultimo, NSW 2007, Australia; 4 Faculty of Medicine, Nursing and Health Sciences, Monash University, 27 Rainforest Walk, Clayton, VIC 3168, Australia; 5 School of Medicine, The University of Notre Dame Australia, 160 Oxford St, Darlinghurst, NSW 2010, Australia

**Keywords:** melanoma, skin, skin cancer, lymph nodes, adjuvant radiotherapy, review

## Abstract

Adjuvant radiation therapy (ART) for macroscopic regional nodal cutaneous melanoma has evolved. A significant step was the discovery of targeted therapies, particularly towards V600E-mutated melanoma, and immunotherapy under its different kinds. Prior to this, the defining trial was the Australia and New Zealand Melanoma Trials Group (ANZMTG) 01.02/Trans-Tasman Radiation Oncology Group (TROG) 02.01 phase III trial that showed that ART using three-dimensional conformal radiotherapy (3DCRT) decreased in-field recurrence by 50% (48 Gray (Gy) in 20 fractions). After the advent of systemic therapies, a trial of the combination targeted therapy of dabrafenib plus trametinib toward BRAF V600-mutant nodal cutaneous melanoma showed that all 35 patients achieved a pathological response. Radiotherapy (RT) was found to be safe to give with concurrent combination therapy. A retrospective immunotherapy study found that in 71 patients that received ART after a first recurrence, further in-field recurrence significantly decreased (*p* = 0.01). For those tumours that do recur in-field, there are now competing therapies like Talimogene laherparepvec or T-VEC. Generally, ART is now used at the first recurrence. The challenge now is to find which melanomas are truly radiosensitive if ART is to have any future role in this scenario.

## Simple Summary

Adjuvant radiation therapy (ART) for macroscopic regional nodal cutaneous melanoma is radiotherapy (RT) that is given after surgery to ensure that the nodal melanoma does not recur in the irradiated field. Prior to the discovery of effective drugs for melanoma, a high-quality trial showed that ART decreased in-field recurrence by 50%. After the advent of effective drugs, nodal melanoma is now treated with these drugs upfront, while local therapies like surgery and ART are reserved for nodal disease that progresses despite the drugs. RT has been found to be safe in this scenario. There are now competing therapies like Talimogene laherparepvec (T-VEC) for local progression. The challenge now is to find which melanomas are truly radiosensitive if ART is to have any future role.

## 1. Introduction

Cutaneous melanoma is increasing in incidence worldwide. The natural history of melanoma is that it arises in usually sun-exposed skin and then metastasizes to drain regional lymph nodes and is called stage III disease. Melanoma then metastasizes to distant organs, particularly the brain, and is called stage IV disease. Some melanomas metastasize to distant structures, bypassing the regional nodes. A certain percentage of melanomas present as already nodal or as a distant disease with an unknown primary. Those with a distant disease can have quite a good performance status, and the diagnosis of stage IV disease at presentation can come as quite a surprise.

There has been rapid progress in all areas of melanoma prevention, detection, staging and treatment over the last few decades based on excellent international multidisciplinary cooperation in high-quality trials. More sensitive diagnostic tests mean that there can be a stage shift. Stage III disease diagnosed only as such by computed tomography (CT) scanning in earlier trials may have really been stage IV when being screened for enrolment. Later, more sensitive tools have become available to prevent this, but this phenomenon of stage shift can make trial comparison over time problematic.

Using baseline total body imaging with positron emission tomography (PET) and magnetic resonance imaging of the brain (MRI brain) for initial staging in high-risk disease has led to the identification of patients who present with already stage IV disease and, rarely, those with other diseases (e.g., another primary malignancy or tuberculosis) that may impact therapy.

Macroscopic regional lymph nodes can be defined as those nodes detected by patients with symptoms and/or detected upon routine physical examination (PE) and/or detected by appropriate imaging. The detection of nodal melanoma has been helped by better surgical and imaging techniques (such as PET and sentinel lymph node biopsy (SLNB)). PET can (i) detect macroscopic regional lymph nodes that may be missed or are beyond routine PE (e.g., the skull base or pelvic nodes), as well as (ii) help in total-body staging to exclude stage IV disease. SLNB detects involved lymph nodes before they become macroscopic. SLNB allows better prognostication and has been pivotal in decreasing the role of completion lymph node dissection (CLND) with its accompanying toxicities. The role of SLNB and the use of RT (in definitive, neo-adjuvant, salvaging, and/or palliative roles) in the treatment of macroscopic, regional lymph nodes will not be discussed in this review.

Melanoma treatment advances have been truly astonishing in the past 20 years. Melanoma has served as a paradigm cancer in how cancer therapy can benefit from multidisciplinary and translational care. The main advances have been in the discovery of targeted therapies, particularly towards V600E-mutated melanoma and immunotherapy under its different kinds, all of which are discussed in more detail in other parts of this series.

Based on the entry of these new therapies into the clinic, there has truly been an evolution of care for those suffering with macroscopic regional nodal melanoma. The rise in systemic therapies in this scenario has been matched by a decrease in the use and intensity of local and regional therapies like surgery and radiotherapy (RT). This change in practice has led to better outcomes. The purpose of this review is to outline this evolution.

## 2. Adjuvant Radiation Therapy in Macroscopic Regional Nodal Melanoma Prior to Effective Systemic Therapy for Melanoma

The internationally recruiting ANZMTG 01.02/TROG 02.01 randomised phase III trial was the defining trial in this scenario [1]. A total of 248 patients who had undergone lymphadenectomy for a palpable lymph-node field relapse (of either a parotid and cervical, axilla, or groin type) and were at a high risk of recurrence were randomly assigned to either adjuvant radiotherapy (ART) or observation (OBS) and analysed.

The trial design stratified by the institution, areas of the lymph-node field (parotid and cervical, axilla, or groin), number of involved nodes (≤3 vs. >3 nodes), maximum involved node diameter (≤4.0 cm vs. >4.0 cm), and extent of extracapsular extension (ECE) (none, limited, or extensive). Trial participants were not masked to treatment allocation, but they were unaware of each other’s treatment allocation. Study outcomes were assessed every quarter from randomisation in the first 2 years, then every 6 months in years 3 to 5, and then annually.

**Figure 1:**
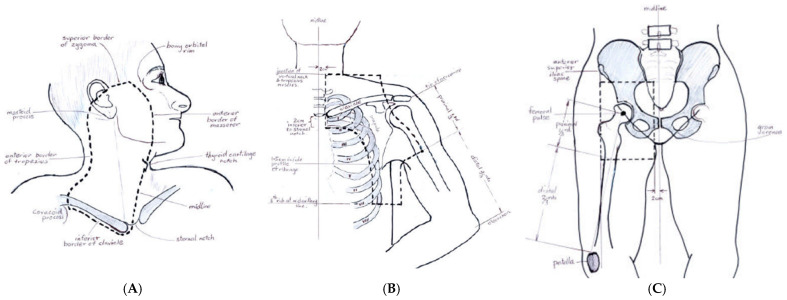
ANZMTG 01.02/TROG 02.01 protocol definitions of the treatment fields used. (**A**) Parotid/cervical electron field. (**B**) Axillary field. (**C**) Groin field.

The RT was given by three-dimensional conformal radiotherapy (3DCRT) of 48 Gray (Gy) in twenty fractions, given at five fractions per week, with maximum overall treatment time of 30 days. See Figure 1 for the definition of the treatment fields. CT planning was used. The tumour dose of 48 Gy was prescribed at the maximum dose point, and the isodose covering the planning target volume (PTV) [2] was to receive 45 Gy. Junctional maximum doses were not to exceed 55 Gy.

The primary endpoint was lymph-node field relapse as a first relapse, assessed in patients without major eligibility infringements (determined by an independent data monitoring committee). Late adverse effects, defined as occurring over 90 days after surgery or the start of radiotherapy, were assessed. Accrual was between 21 March 2003 and 15 November 2007, well before any modern systemic therapies where being used for melanoma. A total of 109/123 patients in the ART arm and 108/127 OBS patients were eligible for efficacy assessments. The last patient (follow-up) visit was 15 November 2011. The median follow-up was 73 months (IQR 61–91).

**Figure 2:**
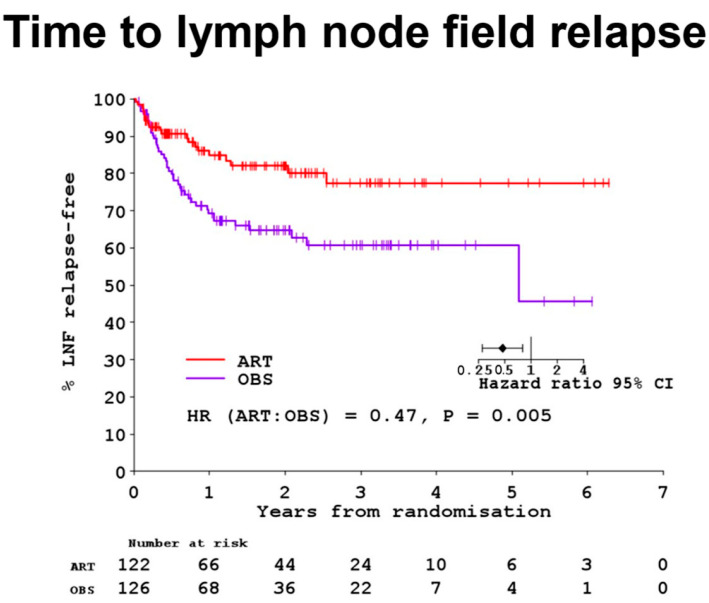
Results of the ANZMTG 01.02/TROG 02.01 randomised trial for the primary endpoint of in-field control. This trial is from the era prior to effective systemic theories for melanoma. This graph shows that adjuvant radiotherapy (ART) significantly improves nodal field control in melanoma patients after lymphadenectomy for macroscopic regional lymph nodes.

The primary endpoint of in-field failure showed that 23 (21%) relapses occurred in the ART group compared to 39 (36%) in the OBS group (adjusted hazard ratio [HR] 0.52 [95% CI 0.31–0.88]; *p* = 0.023). The overall survival (HR 1.27 [95% CI 0.89–1.79]; *p* = 0.21) and relapse-free survival (0.89 [0.65–1.22]; *p* = 0.51) did not differ between the ART and OBS groups. See Figure 2, which shows in graph form the primary endpoint of this trial.

Minor, long-term ART side effects were common and predominantly included pain and fibrosis in the skin or subcutaneous tissue. Twenty (22%) patients receiving ART developed grade 3–4 side effects. Eighteen (20%) patients had grade 3 side effects (mainly affecting the skin (nine [10%] patients) and subcutaneous tissue (six [7%] patients)).

Results over 5 years noted a significant increase in the lower limb volumes after ART to the groin (mean volume ratio of 15.0%) compared to the observation (7.7%; difference of 7.3% [95% CI 1.5–13.1]; *p* = 0.014). There were no significant differences in the upper limb volumes noted between the ART and OBS groups.

In summary, this trial showed that ART at this dose level decreased the regional recurrence of fully resected macroscopic regional nodal melanoma by 50%. This trial was conducted prior to the current era in which effective systemic therapy (such as targeted therapies, i.e., BRAF, MEK, and adjuvant immunotherapy) are now available. In the current era, this trial is useful for informing treatment for those unable to have effective systemic therapy for melanoma.

Currently, better radiation modalities (such as intensity-modulated radiotherapy and its variants, such as volumetric modulated arc therapy) would be used in the radical setting. There have been improvements in how to contour nodal volumes specifically for skin cancer [3]. Trials in other diseases have shown at this dose level that the late toxicity suffered by surrounding normal tissues would hopefully be less than in the 3DCRT era [4].

## 3. Adjuvant Radiation Therapy in Macroscopic Regional Nodal Melanoma in the Era of Effective Systemic Therapy for Melanoma

Unfortunately, none of the adjuvant systemic therapy landmark trials report factors known to be associated with the risk of locoregional recurrence in any detail (specifically the presence of ECE). This limits the ability to directly compare the impact of ART and systemic therapy on the risk of locoregional relapse. Historically, the standard approach to patients with melanoma with the involvement of the sentinel lymph node (SLN) was to proceed to CLND. However, two trials have since demonstrated no improvement in survival in those patients who proceed to CLND (after a positive SLN), and this approach is therefore no longer routinely recommended [5,6,7]. As surgery was not performed, no post-operative histopathology report was available. The latter would have detailed the presence of any high-risk features that may have swayed the multidisciplinary discussion to offer ART. The decreased use of CLND has coincided with a decrease in the use of ART.

### 3.1. Targeted Therapy

Dabrafenib is an inhibitor of some mutated forms of BRAF kinases, including BRAF V600E, often found in melanoma. Trametinib is a reversible inhibitor of MEK1 and MEK2. The combination of these two drugs generates a blockade point in the MAPK pathway at two different levels, inhibiting oncogenic downstream signalling and causing cell cycle arrest in drugs with the relevant mutations.

This combination targeted therapy of dabrafenib plus trametinib toward BRAFV600-mutant melanoma has activity in nodal cutaneous melanoma. Long et al. conducted a single-arm, open-label, single-centre phase 2 study [8]. They assessed the proportion of patients (with histologically confirmed, resectable Response Evaluation Criteria in Solid Tumors (RECIST) with measurable, clinical stage IIIB–C [9] and BRAFV600-mutant melanoma) who had a pathological response after therapy with neoadjuvant dabrafenib plus trametinib. The primary outcomes were the proportion of patients who achieved a complete pathological response and the proportion of patients achieving a RECIST measurable response at week 12 [10].

Between 20 August 2014 and 19 April 2017, 40 patients were screened, of which 35 eligible patients were enrolled into the trial. The median follow-up interval was 27 months (IQR 21–36). After resection and pathological evaluation, all 35 patients achieved a pathological response, of whom 17 (49%; 95% CI 31–66) had a complete pathological response and 18 (51%; 95% CI 34–69) had a non-complete pathological response. Treatment-related serious adverse events occurred in six (17%) of the thirty-five patients; of these thirty-five, ten (29%) patients had grade 3–4 adverse events. No treatment-related deaths were reported. This success of targeted therapy has further reduced the role of adjuvant RT in macroscopic regional nodal melanoma.

If RT is needed during targeted therapy, the question may be asked of whether the combination is safe. Wang et al. conducted a dose-escalation, phase 1 trial evaluating BRAF inhibitors and palliative RT to see if there was as associated increase in toxicity (especially skin toxicity [11]). A total of 10 patients with BRAF-mutant metastatic melanoma treated with concurrent dabrafenib and trametinib and palliative RT (to either soft tissue or nodal or bony metastases) were studied. Side effects and clinical photographs of the irradiated area were collected for up to 12 months following completion of the RT. Six patients were treated at level 1 (20 Gy in five fractions at any location) and four patients at level 2a (30 Gy in ten fractions with no abdominal viscera exposed). All patients completed 12 months of post-RT follow-up. Of the 82 adverse events reported, the majority (90%) were grade 1 and 2. Eight (10%) grade 3 adverse events occurred in five patients (and only one was treatment-related (grade 3 fever due to dabrafenib and trametinib)). No patients experienced grade 3 or 4 RT-related side effects, including no skin toxicities. Wang et al. concluded that targeted therapy may be continued concurrently during fractionated non-visceral palliative RT to extracranial sites. This safety profile was also shown in a retrospective case series from another institution [12].

### 3.2. Immunotherapy

For those unable to have targeted therapy, or who have progressed through it, immunotherapy has been used. Bhave et al. studied the role of ART in patients with melanoma who have resected macroscopic locoregional recurrences during or after adjuvant immunotherapy, including those that may or may not have undergone the prior CLND of the nodal basin [13]. This trial evaluated the rate of locoregional disease control provided by ART in this population.

In this retrospective study, 71 patients with resected stage III melanoma who had received adjuvant anti-programmed cell death protein-1 (PD-1) (±ipilimumab) immunotherapy with a subsequent locoregional (lymph node and/or in-transit metastases) recurrence were identified. At diagnosis, 42 (59%) were men, 30 (42%) had BRAF V600E-mutant melanoma, and 43 (61%) had stage IIIC melanoma. The median time to the first recurrence was 7 months (1–44), at which 24 (34%) received ART and 47 (66%) did not. A total of 49 (69%) developed a melanoma recurrence while still receiving adjuvant immunotherapy.

In this study, patients who received ART had a higher frequency of poor prognostic markers (including the involvement of surgical margins (25% vs. 4%; *p* = 0.01), ECE (61% vs. 33%; *p* = 0.28), and stage IIID disease (17% vs. 6%; *p* = 0.38)). Patients who recurred during adjuvant immunotherapy were more likely to receive ART (75% vs. 66%; *p* = 0.61). Importantly, 75% of patients who received ART at the first recurrence had nodal (+/− in-transit) disease, compared to only 32% that did not receive ART (*p* = 0.0016).

The primary outcome of this trial was the rate of subsequent locoregional melanoma recurrence. A total of 33 patients (46%) developed a second recurrence at a median of five months (1–22). The rate of locoregional relapse at the second recurrence was lower in those patients who had received ART (8%; 2/24) compared to those patients who did not (36%, 17/47; *p* = 0.01).

The secondary outcomes of this trial were locoregional recurrence-free survival (lr-RFS2) and the overall RFS (RFS2) to the second recurrence. ART at the first recurrence was associated with an improved lr-RFS2 (HR 0.16; *p* = 0.015), with a trend towards an improved RFS2 (HR 0.54; *p* = 0.072). The results of this trial showed that there was no effect on risk of distant recurrence or overall survival.

The authors concluded that ART in this scenario was associated with an improved lr-RFS2, with no increased risk of distant recurrence or overall survival. The effect may have been greater given that the higher-risk disease was more likely in this retrospective study to be treated with RT.

The advances in the use of effective systemic therapies for melanoma has meant that ART has now been reserved for patients following the surgical salvage of recurrences with a high risk of a second recurrence post-failure after these therapies.

### 3.3. Progression Post Immunotherapy

Patients can progress with nodal melanoma during or following immunotherapy treatment. A salvaging- or palliative-intent RT may then be used. However, there are currently other competing treatments, for example the use of injected anti-melanoma viruses (one of these is Talimogene laherparepvec, also known as T-VEC [14]). T-VEC is now approved for the treatment of (unresectable, recurrent) melanoma for patients who have progressed on from systemic immunotherapy. The primary outcome of the study by Carr et al., who studied 112 patients with median follow-up of 14 months, was an in-field response of 37% with a complete response and 14% with a partial response. The median in-field progression-free survival (PFS) was 15 months, and the median overall disease-free survival (DFS) after CR was 32 months. Another earlier smaller retrospective study by Fröhlich et al. found similar results [15]. A prospective study using RT would need to produce similar results for RT to be considered a competing modality to T-VEC today.

## 4. Discussion

This evolution of continually improving patient care continues on the basis of high-quality clinical trials and global collaborations among those who can treat patients. It is important to appreciate that these improvements are based on multidisciplinary care involving an ever-increasing number of specialities and craft groups. Success depends on a commitment to clear communication between all the personnel involved and putting patient outcomes before modality survival.

The history of how the treatment of macroscopic regional nodal melanoma has changed could also be a paradigm for how medical leadership can change from what was a scenario with a heavy reliance on surgical and radiotherapy modalities to a scenario now dominated by systemic therapies. The transition has been quite seamless, which is a credit to the craft groups involved.

The downside of this evolution is that those who cannot have systemic therapies or who have progressed through them still need to be salvaged by what were the dominant therapies of yesteryear, that is, salvage surgery and radiotherapy.

Just as medical oncologists have found subtypes of melanoma that are more sensitive to some systemic therapies than others (e.g., BRAF-mutant), the challenge for radiation oncologists is to find which melanomas are more radiosensitive than others and why. Clinical experience shows that melanomas have different radiation sensitivities. Discovering the scientific basis of this and how to test for it is the challenge. Some think that it could be due to genetic mutations; others even think that it is due to different concentrations of trace metals [16]. More research is needed.

## 5. Conclusions

ART for macroscopic regional nodal cutaneous melanoma has evolved over time, as have other modalities involved with patients suffering from this disease. This article describes how the discovery and implementation in the clinic of effective systemic therapies have replaced surgery and ART as first-line treatment in this scenario. This has all been based on high-quality studies. It is a tribute to those involved in caring for these patients that this transition has happened so seamlessly. Radiotherapy is now really reserved for those who decline or who are unable to tolerate or have progressed through effective systemic therapies. There are now other options for local therapies to deal with nodal metastases. The challenge for radiation oncologists is to find what types of melanoma are radiation-sensitive.

## Data Availability

All the data quoted is available in the public domain via the references.
